# Occurrence and characterization of methicillin-resistant *Staphylococcus* spp. in diseased dogs in Brazil

**DOI:** 10.1371/journal.pone.0269422

**Published:** 2022-06-03

**Authors:** Flávia Mello Viegas, Jordana Almeida Santana, Brendhal Almeida Silva, Rafael Gariglio Clark Xavier, Cláudia Teixeira Bonisson, Júlia Lara Sette Câmara, Mário Cesar Rennó, João Luis Reis Cunha, Henrique César Pereira Figueiredo, Francisco Carlos Faria Lobato, Rodrigo Otávio Silveira Silva

**Affiliations:** 1 Veterinary School, Federal University of Minas Gerais, Belo Horizonte, Minas Gerais, Brazil; 2 VetMaster Veterinary Clinic, Belo Horizonte, Minas Gerais, Brazil; 3 Department of Biology, University of York, Heslington, York, United Kingdom; Universidade de Lisboa Faculdade de Medicina, PORTUGAL

## Abstract

*Staphylococcus pseudintermedius* is a major commensal bacterium of the skin and mucosae of dogs and an opportunistic agent responsible for several clinical infections, such as pyoderma, otitis, and surgical wound infections. The emergence of methicillin-resistant *S*. *pseudintermedius* (MRSP) has become a problem of great concern in veterinary and human medicine because it is multidrug resistant (MDR) and can also infect humans. This study aimed to identify the occurrence of *Staphylococcus* spp. in infected patients and investigate the antimicrobial resistance profiles and molecular structure of MRSP isolates. Samples were obtained from two different veterinary clinics; suggestive colonies were submitted to matrix-assisted laser desorption ionization-time of flight (MALDI-ToF) mass spectrometry and confirmed at the species level by polymerase chain reaction (PCR). Sequencing of the *16S rRNA* and *rpoB* genes were used in selected samples that were not identified by MALDI-ToF and by the species-specific PCR. Antimicrobial susceptibility and PCR detection of *mecA* were performed. MRSP isolates were subjected to multilocus sequence typing. Of all the clinical staphylococci (n = 131), 98 (74.8%) were identified as *S*. *pseudintermedius*. Multidrug resistance (resistance to ≥3 classes of antimicrobials) was observed in 63.2% of *S*. *pseudintermedius* isolates, and 24.5% of *S*. *pseudintermedius* isolates were methicillin-resistant. Half of the MRSP isolates were isolated from surgical site infections. Among the ten sequence types (ST) identified, nine were novel. ST71 was the most prevalent and associated with resistance to fluoroquinolones. Prior antimicrobial therapy, hospitalization, and surgical site infections seemed to be risk factors for MRSP acquisition. The present study showed a high rate of MDR staphylococci in infected dogs. MRSP was isolated from different clinical conditions, mainly surgical site infections. Additionally, this is the first study to extensively investigate the population structure of MRSP in Brazil, which revealed the dispersion of CC71 and nine novel ST. These findings raise concerns for both animal and human health due to the zoonotic potential of this species and limited therapeutic options available for MRSP infections.

## Introduction

*Staphylococcus pseudintermedius* is a major commensal agent of the skin and mucosae of dogs and integrates the *S*. *intermedius* group (SIG) along with three other species: *S*. *intermedius*, *S*. *delphini* and *S*. *cornubiensis* [1, 2]. Although it is commonly found in the nose and anus of healthy individuals, *S*. *pseudintermedius* can be responsible for several clinical infections, being one of the leading causes of pyoderma, but it is also a common cause of otitis, wound infections, and urinary tract infections [1, 3–5].

Over the past decade, the emergence of methicillin-resistant *S*. *pseudintermedius* (MRSP) has become a problem of great concern in veterinary medicine [4, 6, 7] due to the acquisition of the Staphylococcal Cassette Chromosome *mec* (SCC*mec*), a mobile genetic element which harbors the *mecA* gene and confers resistance to all beta-lactam antimicrobials, except for the fifth-generation cephalosporins ceftaroline and ceftobiprole [8]. MRSP strains are also commonly resistant to other antimicrobial classes, such as aminoglycosides, macrolides, and fluoroquinolones, representing a challenge to therapeutic approaches [4, 9–11]. Furthermore, SCC*mec* and/or other antimicrobial resistance genes can be transferred between different staphylococci, which can lead to serious public health issues, especially when involving MRSP and methicillin-resistant *S*. *aureus* (MRSA) [9, 12, 13]. Similar to MRSP in dogs, MRSA is a major pathogen associated with both hospital and community-acquired infections in humans and is responsible for severe cases of soft tissue infections [11, 12].

Reports of human cases of carriage or infection by *S*. *pseudintermedius* have been scarcely described over the last few years [9, 14]. *S*. *pseudintermedius* can cause a range of skin and soft tissue infections in humans, and studies have shown high similarity among strains isolated from human patients and their dogs [15–19]. Furthermore, it seems like long-term contact with dogs may be a risk factor for acquiring the bacterium, with veterinarians and pet owners being the most affected [9, 14, 20, 21]. In this context, MRSP is a relevant one health problem because of its potential zoonotic risk and the limited options of treatment currently available.

In addition to its growing importance, there are few studies on MRSP in Brazil. To date, there is almost no information about its population structure and resistance patterns [22–25]. Furthermore, few studies have investigated the possible relationship between different clinical manifestations caused by *S*. *pseudintermedius* in dogs and their patterns of antimicrobial resistance (AMR) and genetic background. This study aimed to identify the occurrence of staphylococci, with major focus on *S*. *pseudintermedius*, in dogs with clinical conditions and investigate its epidemiology in Belo Horizonte, Minas Gerais, by reporting the antimicrobial resistance profiles and analyzing the molecular structure of MRSP isolates.

## Materials and methods

### Strain isolation and identification

Clinical specimens were obtained from dogs during consultation at the Veterinary Hospital of the Federal University of Minas Gerais (HV-UFMG) and in a veterinary clinic (VetMaster, Belo Horizonte, Minas Gerais) from 2017 to 2020. After informed consent of the owners, samples of dogs with suspected bacterial infection at any site were included in the present study. Patient information data, including reasons for consultation, age, sex, disease, specimen type, involvement of antimicrobial therapy at the time of sampling, and outcome, were collected. This study was approved by the Ethical Committee on Animal Use (CEUA) of the Federal University of Minas Gerais under protocol 287/2019.

All samples were plated on mannitol salt agar (Kasvi, Brazil) and incubated for 24 h at 37°C. Two presumptive colonies from each sample were plated on Müller Hinton agar (Difco, USA) and identified by matrix-assisted laser desorption/ionization time-of-flight (MALDI-TOF) mass spectrometry, as described previously [26]. Briefly, for each strain, 1 μl of formic acid (70%) and 1 μl of MALDI-TOF MS matrix, consisting of a saturated solution of α-cyano-4-hydroxycinnamic acid (HCCA) (Bruker Daltonics, Bremen, Germany), were applied to the spot and allowed to air-dry. Spectra were acquired using the FlexControl MicroFlex LT mass spectrometer (Bruker Daltonics) with a 60-Hz nitrogen laser, in which up to 240 laser shots. Parameters for mass range detection were defined to allow the identification from 1,960 to 20,137 m/z, where Ion source 1 v was 19.99 kv, Ion source 2 voltage was 18.24 kv and the lens voltage was 6.0 kv for data acquisition. Prior to measurements, calibration was preceded with a bacterial test standard (*E*. *coli* DH5 alpha; Bruker Daltonics). The Real Time (RT) identification score criteria used were those recommended by the manufacturer: score ≥ 2.000 indicates a species-level identification. Isolates identified as SIG by MALDI-ToF were confirmed by multiplex polymerase chain reaction (PCR) of *nuc* gene [27]. SIG isolates that were not amplified by multiplex PCR, and non-SIG isolates with MALDI-ToF score under 2.000 were submitted to sequencing of the *16S rRNA* gene and sequencing of the *rpoB* gene, if necessary. Amplification and sequencing of the *16S rRNA* and *rpoB* genes were performed as previously described [28, 29].

### Antimicrobial susceptibility testing

Antimicrobial susceptibility of all *Staphylococcus* spp. isolates was tested by the disk diffusion method and interpreted according to the Clinical and Laboratory Standards Institute (CLSI) documents, M100-Ed31 [30] and VET01S-Ed5 [31]. Strains were cultivated in Müller-Hinton agar and incubated overnight at 35° C. Inoculation was performed using cell suspensions of 0.5 McFarland turbidity standard and results were read within 18 to 24 hours. The following antimicrobials were tested: oxacillin (OXA, 1 μg), cefoxitin (FOX, 30 μg), penicillin (PEN, 10 IU), gentamicin (GEN, 10 μg), erythromycin (ERY, 15 μg), clindamycin (CLI, 2 μg), tetracycline (TET, 30 μg), ciprofloxacin (CIP, 5 μg), nitrofurantoin (NIT, 300 μg), trimethoprim-sulfamethoxazole (SXT, 1,25/23,75 μg), chloramphenicol (CHL, 30 μg), and rifampicin (RIF, 5 μg) (Oxoid, USA). *Staphylococcus aureus* ATCC® 25923 was used as control. The results were recorded as susceptible, intermediate, or resistant by measuring the inhibition halo diameter according to the documents quoted above [30].

### DNA extraction and detection of *mecA*

Extraction of bacterial genomic DNA with guanidium thiocyanate was performed according to a method previous described [32]. The extracted DNA was quantified using a Nanodrop spectrophotometer (Thermo Scientific, Wilmington, DE, USA). The purity of the extracted DNA was determined using the absorbance ratio at 260/280 nm. All staphylococci isolates were tested by PCR to determine whether they carry the *mecA* gene [33].

### Multilocus sequence typing (MLST)

Multilocus sequence typing was performed for all MRSP isolates, according to literature [34, 35]. STs were determined using the *S*. *pseudintermedius* MLST database (https://pubmlst.org/organisms/staphylococcus-pseudintermedius), and new STs were assigned by the curator Vincent Perreten (vincent.perreten@vbi.unibe.ch). Phyloviz v 2.0, using the goeBURST algorithm [36, 37], was used to infer the population structure, with clonal complexes (CCs) composed of all strains sharing at least six identical alleles (single-locus variant). Multilocus sequence analysis (MLSA) was performed based on concatenated sequences of the seven alleles from the MLST scheme. A phylogenetic tree was constructed using the approximately-maximum-likelihood (ML) model and Hasegawa-Kishino-Yano models of nucleotide evolution was constructed using IQ-TREE [38], with a bootstrap analysis with 1000 replicates, and visualized and annotated using iTol v.4 [39].

### Statistical analysis

Data were summarized using frequency tables and percentages for categorical variables and medians for continuous variables. To measure the association between the categorical variables and isolated strains, a univariate analysis using Fisher’s exact test was performed. The associations were expressed as odds ratios (ORs) and their 95% confidence intervals (CIs). Statistical significance was set at *P* ≤ 0.05. For quantitative variables, the Mann-Whitney test was used, with a *p* value ≤ 0.05, and Tukey’s comparison test was applied if statistical significance was observed. All analyses were performed using R Software 4.0.9 (R Development Core Team, NZ).

## Results

### Data collection

One hundred and sixteen dogs were included in this study, which provided 131 samples of staphylococcal species. When two isolates from the same animal were obtained from the same site and had the same antimicrobial resistance profile, only one of them was maintained in the study. Most of the isolates (95/131–72.5%) were obtained from HV-UFMG, while 27.5% (36/131) samples from 27 animals were obtained in a private clinic. The animals belonged to different breeds and ages ranging from 12 to 204 months, with a mean of 88 months (±46.3). Fifty-seven animals were female, fifty-eight were male, and one was not provided with gender information (see S1 File). At least 64 animals (55.2%) had previous use of antimicrobials, 56 received different systemic antibiotics, eight of them had used only topical antibiotics, and 15 of them used both systemic and topical products. The main prescribed antibiotics were cefalexin (29/64), amoxicillin (9/64), or a combination of amoxicillin and clavulanic acid (16/64) and enrofloxacin (11/64). Most of the clinical specimens were associated with dermatological conditions: 58 (44.3%) with pyoderma, 28 (21.4%) with otitis, 20 (15.3%) with urogenital tract infections, 15 (11.4%) with surgical site infections (SSIs), and 10 (7.6%) from other sites.

### Strain isolation and identification

**Fig 1:**
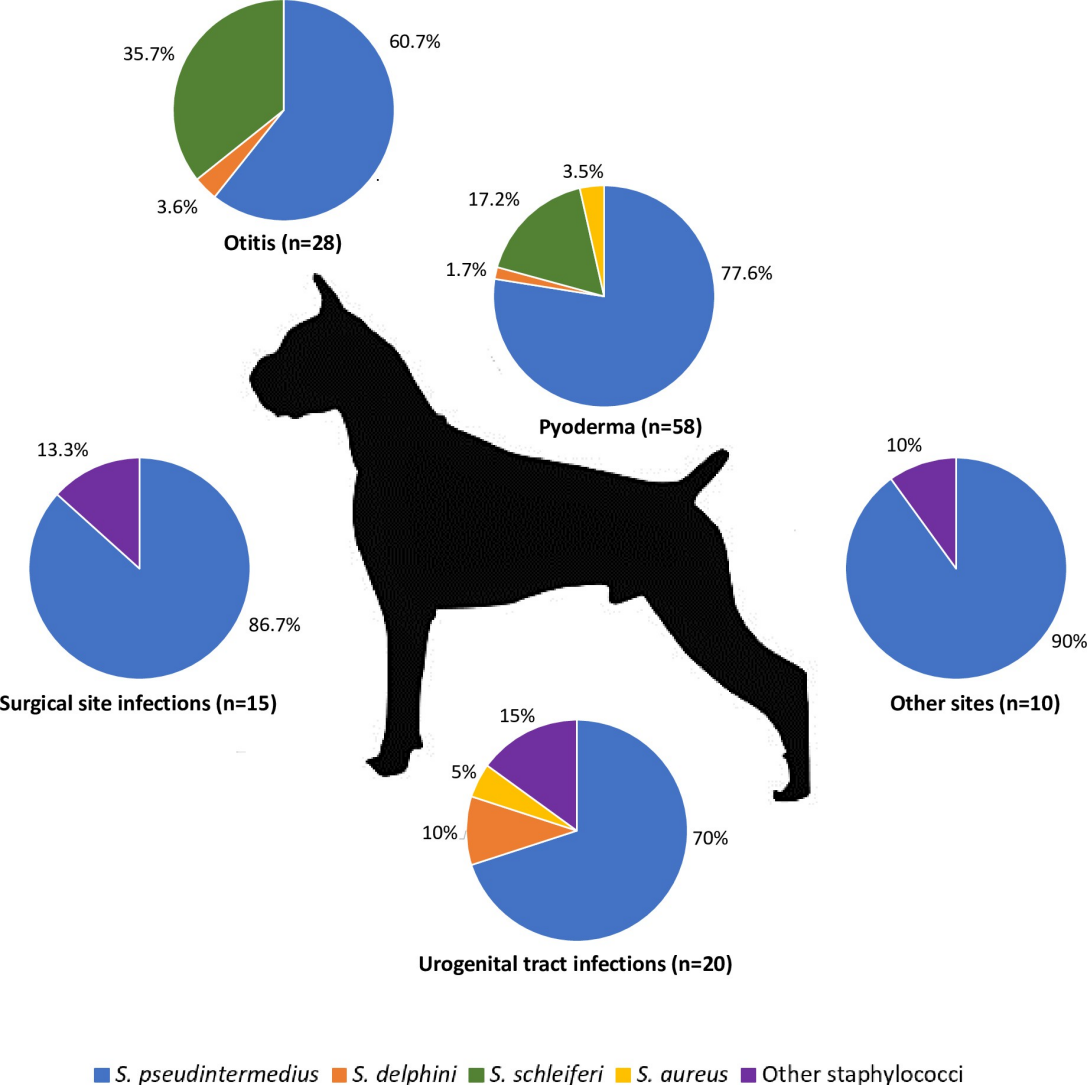
Distribution and proportion of staphylococci from different sites of sampling from 116 dogs attended at two different veterinary clinics from Belo Horizonte, Brazil, between 2017 and 2020. Disclosure—The recently approved differentiation of *S*. *schleiferi* into two species, *S*. *coagulans* and *S*. *schleiferi* [40], was not considered in the present work.

A total of 131 samples were obtained and confirmed to be *Staphylococcus* spp. by MALDI-ToF. A total of 102 isolates (77.9%) were identified by MALDI-TOF as part of the SIG (Table 1). Among these, PCR of the *nuc* gene confirmed 98 (96.1%) isolates as *S*. *pseudintermedius* and four (3.9%) as *S*. *delphini*. Sequencing of the *16S rRNA* gene confirmed all species, except in one case. The only sample sent for sequencing of the *rpoB* gene was identified as *S*. *delphini*, confirming the MALDI-ToF results. The most frequent species isolated was *S*. *pseudintermedius* (98/131, 74.8%), followed by *S*. *schleiferi* (20/131, 15.3%) (see Fig 1), although it is important to state that the possible differentiation of *S*. *schleiferi* into two species, *S*. *coagulans* and *S*. *schleiferi* [40], was not investigated. Nine animals had both pyoderma and otitis at time of sampling–two of them caused by *S*. *pseudintermedius* and two caused by *S*. *schleiferi*; in all those cases, isolates from the same animal showed the same antimicrobial resistance profile. The other five animals had otitis and pyoderma caused by different species, more specifically a combination of *S*. *pseudintermedius* and *S*. *schleiferi* in four of them and the other one with a combination of *S*. *pseudintermedius* and *S*. *delphini*. Two dogs with pyoderma carried methicillin-susceptible *S*. *pseudintermedius* (MSSP) with different patterns of antimicrobial resistance, one carried both MSSP and MRSP and two carried *S*. *pseudintermedius* and *S*. *aureus*.

**Table 1:** Distribution and proportion of staphylococci species isolated from different sites of sampling from 116 dogs at two different veterinary clinics from Belo Horizonte, Brazil, between 2017 and 2020.

Infection site	Species
**Skin (n = 58–44.3%)**	*S*.* pseudintermedius *(n = 45)
	*S*.* delphini *(n = 1)
	*S.* schleiferi *(n = 10)
	*S*. *aureus *(n = 2)
**Ear (n = 28–21.4%)**	*S*.* pseudintermedius *(n = 17)
	*S*.* delphini *(n = 1)
	*S.* schleiferi *(n = 10)
**Urogenital tract (n = 20–15.3%)**	*S*.* pseudintermedius* (n = 14)
	*S*.* delphini *(n = 2)
	*S*. *aureus* (n = 1)
	*S*. *capitis *(n = 1)
	*S*. *epidermidis *(n = 1)
	*S*.* warneri *(n = 1)
**Surgical wound (n = 15–11.4%)**	*S*.* pseudintermedius *(n = 13)
	*S*.* simulans *(n = 1)
	*S*. *capitis *(n = 1)
**Others (n = 10–7.6%)**	*S*.* pseudintermedius *(n = 9)
	*S*.* simulans *(n = 1)

* The recently approved differentiation of *S*. *schleiferi* into two species *(S*. *coagulans* and *S*. *schleiferi*) was not considered in the present work.

### Antimicrobial susceptibility

**Fig 2:**
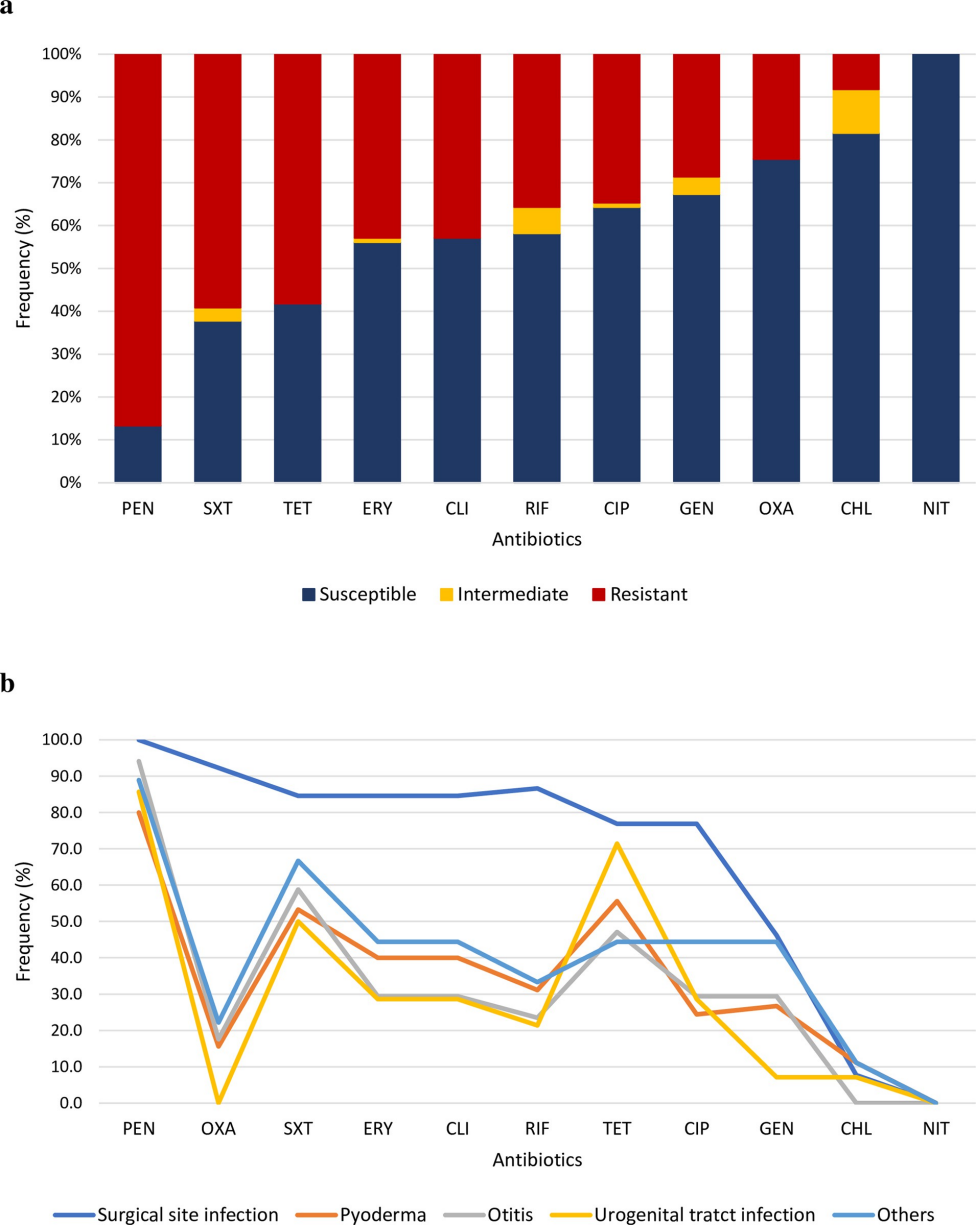
Antimicrobial resistance among clinical *S*. *pseudintermedius* isolates from infected dogs from two different veterinary clinics from 2017 to 2020 in Belo Horizonte, Brazil. **a)** Overall prevalence according to the antimicrobial compound; **b)** According to site of infection.

Results of antimicrobial resistance to all staphylococci species are shown in Table 2. A total of 88.2% (90/102) isolates from the SIG were resistant to at least one antibiotic. About 86.7% (85/98) of *S*. *pseudintermedius* were resistant to penicillin and 24.5% (24/98) were resistant to oxacillin. In sequence, 59.2% (58/98) isolates were resistant to trimethoprim/sulfamethoxazole, 58.2% (57/98) were resistant to tetracycline and 42.9% (42/98) were resistant to clindamycin and erythromycin. All isolates were susceptible to nitrofurantoin. Isolates from SSIs had the highest rates of resistance to nine of the tested antibiotics (p<0.05; Fig 2). Sixty-two isolates (63.3%) were considered multidrug resistant (MDR). Of these, 43.5% (27/62) were isolated from pyoderma, 21% (13/62) from SSIs, 14.5% (9/62) from otitis, 11.3% (7/62) from urogenital infections, and 9.7% (6/62) from other sites. Only one *S*. *delphini* showed antimicrobial resistance, being resistant to penicillin and tetracycline.

**Table 2:** Antimicrobial resistance among staphylococci isolates from infected dogs attended at two different veterinary clinics, from 2017 to 2020, in Belo Horizonte, Brazil.

*Species*	Isolates	OXA/FOX	PEN	ERY	CLI	SXT	RIF	GEN	TET	CIP	CHL	NIT	MDR
***S*. *pseudintermedius***	98 (74.8%)	24 (18.3%)	85 (64.9%)	42 (32.1%)	42 (32.1%)	58 (44.3%)	35 (26.7%)	28 (21.4%)	57 (43.5%)	34 (26%)	8 (6.1%)	0 (0%)	62 (47.3%)
***S*. *delphini***	4 (3,1%)	0 (0%)	1 (0.8%)	0 (0%)	0 (0%)	0 (0%)	0 (0%)	0 (0%)	1 (0.8%)	0 (0%)	0 (0%)	0 (0%)	0 (0%)
***S*. *schleiferi*** *	20 (15.3%)	0 (0%)	2 (1.5%)	0 (0%)	0 (0%)	0 (0%)	0 (0%)	2 (1.5%)	0 (0%)	0 (0%)	0 (0%)	0 (0%)	0 (0%)
***S*. *aureus***	3 (2.3%)	0 (0%)	2 (1.5%)	1 (0.8%)	0 (0%)	0 (0%)	0 (0%)	0 (0%)	1 (0.8%)	0 (0%)	0 (0%)	0 (0%)	0 (0%)
***S*. *capitis***	2 (1.5%)	0 (0%)	2 (1.5%)	0 (0%)	0 (0%)	0 (0%)	0 (0%)	0 (0%)	0 (0%)	0 (0%)	0 (0%)	0 (0%)	0 (0%)
***S*. *epidermidis***	1 (0.8%)	1 (0.8%)	1 (0.8%)	0 (0%)	1 (0.8%)	1 (0.8%)	0 (0%)	0 (0%)	0 (0%)	0 (0%)	0 (0%)	0 (0%)	1 (0.8%)
***S*. *simulans***	2 (1.5%)	0 (0%)	1 (0.8%)	0 (0%)	0 (0%)	0 (0%)	0 (0%)	0 (0%)	0 (0%)	0 (0%)	0 (0%)	0 (0%)	0 (0%)
***S*. *warneri***	1 (0.8%)	0 (0%)	1 (0.8%)	0 (0%)	0 (0%)	0 (0%)	0 (0%)	0 (0%)	0 (0%)	0 (0%)	0 (0%)	0 (0%)	0 (0%)
**Total**	131 (100%)	25 (19.1%)	95 (72.5%)	43 (32.8%)	43 (32.8%)	59 (45%)	35 (26.7%)	30 (22.9%)	59 (45%)	34 (26%)	8 (6.1%)	0 (0%)	63 (48.1%)

OXA = oxacillin, FOX = cefoxitin, PEN = penicillin, ERY = erythromycin, CLI = clindamycin, SXT = thrimethoprim/sulfamethoxazole, RIF = rifampicin, GEN = gentamicin, TET = tetracycline, CIP = ciprofloxacin, CHL = chloramphenicol, NIT = nitrofurantoin, MDR = multidrug resistacnce

* The recently approved differentiation of *S*. *schleiferi* into two species (*S*. *coagulans* and *S*. *schleiferi*) was not considered in the present work.

Among the 29 non-SIG isolates, 18 (62.1%) were susceptible to all antimicrobials. Nine isolates (31%) were resistant to penicillin, which was the highest rate of resistance observed among these strains. Two isolates (6.9% - 2/29) were resistant to GEN and two isolates resistant to PEN were also resistant to ERY (3.4% - 1/29) or TET (3.4% - 1/29). One isolate (3.4% - 1/29) was resistant to FOX, PEN, CLI and SXT and positive for the *mecA*, thus being the only isolate considered multidrug resistant.

*S*. *pseudintermedius* was significantly associated with resistance to RIF (OR 5.82, 95%CI), CIP (OR 5.83, 95%CI), CLI (OR 7.82, 95%CI), ERY (OR 7.82, 95%CI), PEN (OR 10.11, 95%CI), OXA (OR 13.11, 95%CI), SXT (OR 14.73, 95%CI), and TET (OR 14.73, 95%CI). *S*. *pseudintermedius* strains were also associated with MDR (OR 18.6, 95%CI 5.3–65.1) when compared to other staphylococci.

### Methicillin-resistant staphylococci

Approximately 23.5% (24/102) of SIG isolates were positive for the *mecA* gene, all of them identified as *S*. *pseudintermedius*. Three (10.3%) of non-SIG isolates were also positive for *mecA*. Of these, two were *S*. *schleiferi* and one was *S*. *epidermidis*. However, only *S*. *epidermidis* was phenotypically resistant to oxacillin or cefoxitin (inhibition halo = 24mm for FOX); this strain was obtained from a dog with a nosocomial UTI. The two *S*. *schleiferi* positive for *mecA* were not resistant to oxacillin (inhibition halos for FOX 19 and 19,1mm). No MRSA isolates were identified in this study.

### MRSP

Twenty-four isolates of *S*. *pseudintermedius* were resistant to oxacillin and positive for the *mecA* gene by PCR, thus being considered MRSP (Table 3). Of these, 50% (12/24) were from surgical wounds, 29.2% (7/24) from skin lesions, 12.5% (3/24) from otitis, and 8.3% (2/24) from other sites. When classified by the type of infection, rates of MRSP were 15.6% (7/45) in dogs with skin infections, 17.6% (3/17) in dogs with otitis, 92.3% (12/13) in dogs with SSIs, and 22.2% (2/9) in other sites. None of the MRSP strains were isolated from dogs with urinary tract infection (UTI). All isolates were resistant to at least three classes of non-beta-lactam antimicrobials. The most common antimicrobial resistance profiles were OXA-PEN-ERY-CLI-SXT-RIF-CIP-TET-GEN (16.7% - 4/24) and OXA-PEN-ERY-CLI-SXT-RIF-TET-GEN (16.7% - 4/24).

**Table 3:** Antimicrobial resistance profile of methicillin-resistant *S*. *pseudintermedius* (MRSP) strains isolated from dogs (Belo Horizonte, Brazil) according to sequence types (STs) and infection sites.

ST	CC	Antimicrobial resistance profile	Infection site
**71 (n = 9)**	71	OXA, PEN, ERY, CLI, SXT, RIF, CIP, TET, GEN	Surgical wound (2)
			Skin (1)
		OXA, PEN, ERY, CLI, SXT, RIF, CIP, GEN	Surgical wound (1)
			Skin (1)
			Others (1)
		OXA, PEN, ERY, CLI, SXT, RIF, CIP, TET	Surgical wound (3)
**2124 (n = 2)**	NA	OXA, PEN, ERY, CLI, SXT, CIP, GEN	Otitis (1)
		OXA, PEN, SXT, RIF, CIP, TET, GEN	Others (1)
**2125 (n = 3)**	NA	OXA, PEN, ERY, CLI, SXT, RIF, CIP, GEN	Surgical wound (1)
		OXA, PEN, ERY, CLI, SXT, CIP, GEN	Skin (1)
		OXA, PEN, ERY, CLI, SXT, CIP	Surgical wound (1)
**2126 (n = 4)**	NA	OXA, PEN, ERY, CLI, SXT, RIF, TET, GEN	Skin (2)
			Surgical wound (1)
			Otitis (1)
**2127 (n = 1)**	NA	OXA, PEN, SXT, RIF, TET	Skin (1)
**2128 (n = 1)**	NA	OXA, PEN, SXT, RIF, CIP, TET	Surgical wound (1)
**2129 (n = 1)**	NA	OXA, PEN, ERY, CLI, CHL, CIP, TET, GEN	Surgical wound (1)
**2130 (n = 1)**	71	OXA, PEN, ERY, CLI, SXT, RIF, CIP, TET, GEN	Otitis (1)
**2131 (n = 1)**	NA	OXA, PEN, ERY, CLI, SXT, RIF, TET	Surgical wound (1)
**2164 (n = 1)**	NA	OXA, PEN, ERY, CLI, SXT, RIF, CHL, CIP, TET, GEN	Skin (1)

*NA = not applicable.

**Fig 3:**
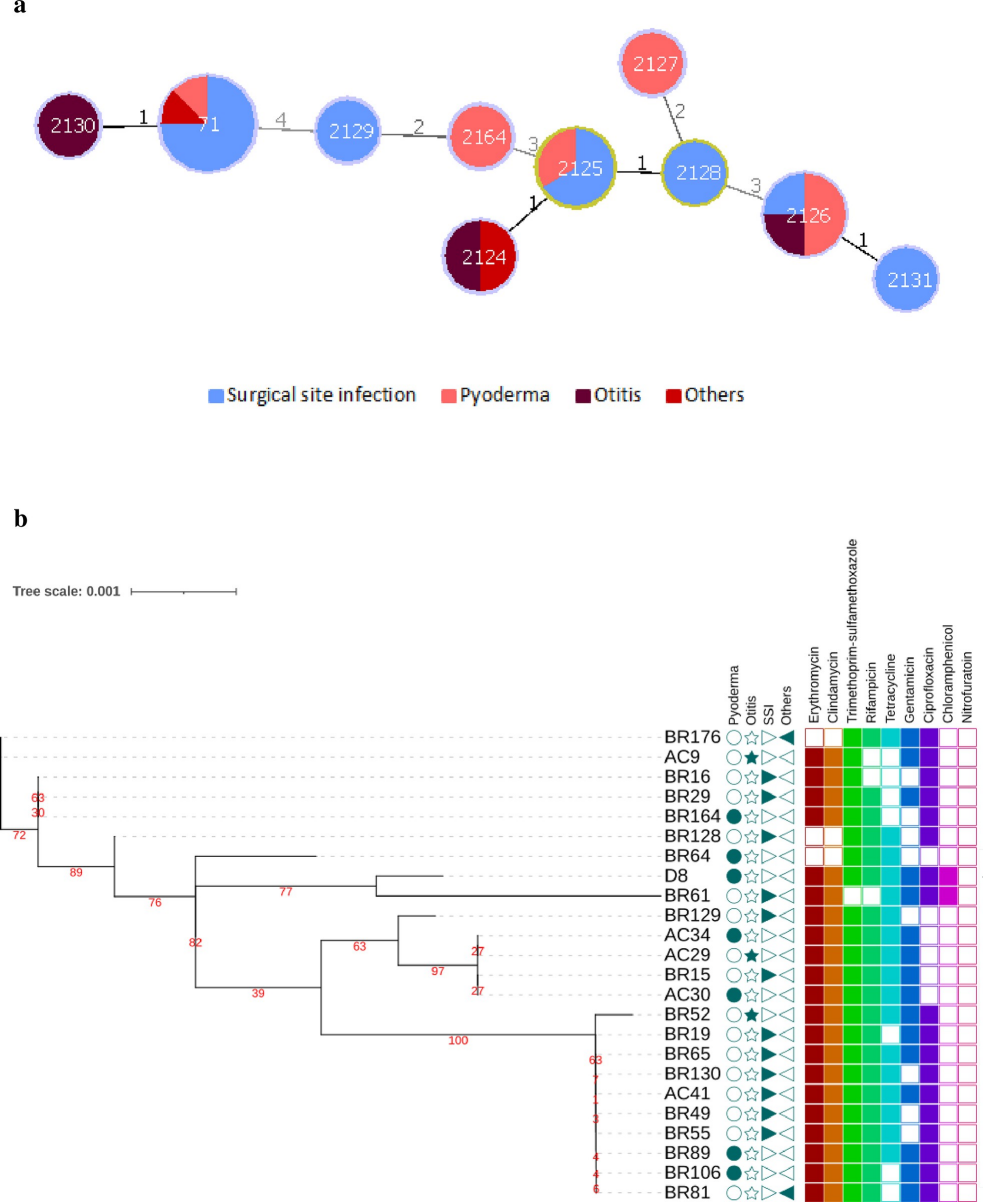
Genetic relationship of MRSP from dogs in Belo Horizonte (2017–2020). **a)** Population snapshot of MRSP goeBURST full MST analysis. Line numbers and shading indicate the number of differing loci between STs and colors indicate the type of infection related to each ST. **b)** A phylogenetic tree based on the alignment of all MLST genes of each ST was inferred using iqTree; posterior probabilities are shown in red. Columns with triangles, circle and star indicate the site of infection of each isolate; colored squares’ columns indicate presence or absence of resistance to different classes of non-beta-lactams antibiotics.

**Fig 4:**
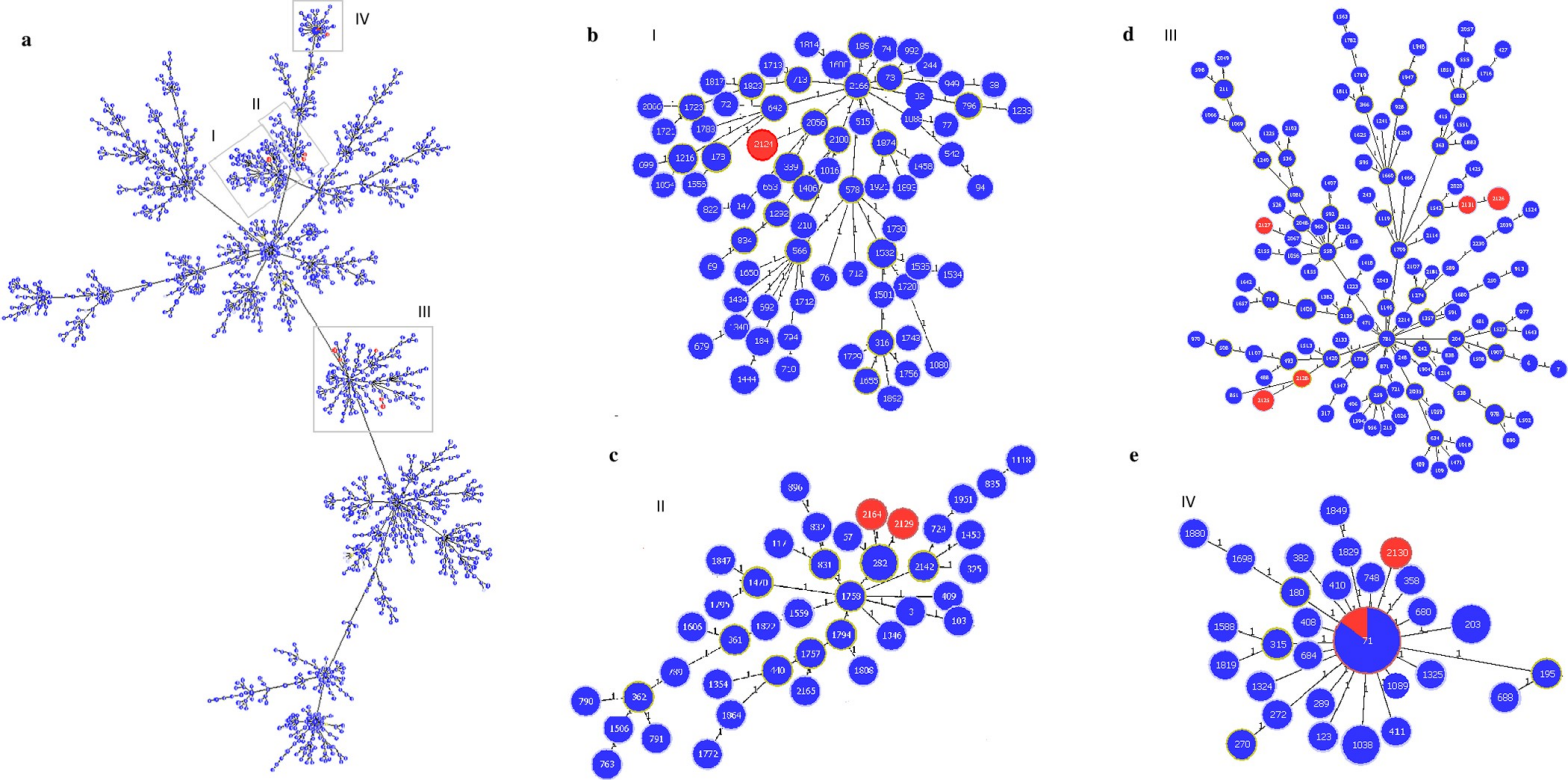
goeBURST population snapshot of *S*. *pseudintermedius* at single locus variance (SLV). Blue color indicates worldwide strain types, while red color indicates MRSP strains identified in the present study; line numbers indicate allelic variance. **a)** Major populational structure of *S*. *pseudintermedius*. Clusters of linked isolates correspond to clonal complexes (CCs); singletons are not showed. Gray boxes indicate the localization of STs identified in the current study. **b)** snapshot of CC2166, with ST 2124 in red. **c)** snapshot of CC1758, with STs 2129 and 2164 in red. **d)** snapshot of CC781 and adjacent, with STs 2125, 2126, 2127, 2128 and 2131 in red. **e)** Snapshot of CC71 with STs 71 and 2130 in red.

Ten different ST profiles were identified, including nine new STs (ST2124 –ST2131 and ST2164) (Fig 3). In goeBURST analysis, ten isolates (ST71 and ST2130) belonged to well-known CC71 (Fig 4E); ST2124 was related to CC2166 (Fig 4B); STs 2125 and 2128 seemed to be related to CC781 (Fig 4D), while ST2127 was closer to CC558 –which has evolved from CC781(Fig 4D); STs 2126 and 2131 were close to subfounder ST1709 (Fig 4D); and STs 2129 and 2164 were closer to CC1758 (Fig 4C). In the statistical analysis, CC71 was associated with resistance to ciprofloxacin (p < 0.05).

### Predictors for MRSP

There was no significant difference in the proportion of MRSP isolates between female and male dogs or between age groups. However, previous antimicrobial treatment (OR9.21 95%CI 2.05–41.25) and hospital-acquired infections were associated with the isolation of MRSP strains. When considering the clinical condition presented by the animals, SSIs were significantly associated with MRSP isolation: a dog with SSI was more likely to carry an MRSP strain than an MSSP strain (OR 3.7 95%CI 1.5–9.4). In addition, MRSP was more likely to be isolated from SSI than from pyoderma, otitis, urogenital infection, or other site infections (p<0.05).

## Discussion

In the present study, we characterized the occurrence of different species of *Staphylococcus* spp. isolated from several different tissues in dogs and evaluated their antibiotic resistance. *S*. *pseudintermedius* was the most prevalent species in this study. As it is the major causative agent of pyoderma in dogs, most clinical specimens were obtained from skin lesions [1, 41]. The second most frequently observed species was *S*. *schleiferi*, but it is important to remember that the recently approved differentiation of *S*. *schleiferi* into *S*. *coagulans* and *S*. *schleiferi* [40] was not considered in the present work. Anyway, *S*. *schleiferi* is rarely found in healthy carriers and is commonly isolated from dogs with otitis or pyoderma [11, 42, 43]. Some animals were infected by both MSSP and MRSP, or even by two different species of *Staphylococcus* spp., similar to previous reports [1, 11]. As these bacteria can share MGE, it is worrisome that strains previously susceptible could acquire resistance and/or virulence genes when in contact with virulent/resistant lineages [10, 12].

High rates of AMR were observed among the SIG isolates to most of the antimicrobials tested in this study. AMR is considered a natural phenomenon, but the excessive or inappropriate use of antimicrobial drugs in human and veterinary medicine accelerates its occurrence [44]. Over half of the animals included in the study had been previously treated with at least one systemic antibiotic, which can be linked to the high rates of AMR found. Unfortunately, this frequency is not surprising, considering the main clinical manifestations presented by those animals. In small animal practice, skin infections are commonly seen in routine, being the main reason for antimicrobial administration in dogs, accounting for almost one-third of antimicrobial prescriptions in small animal clinics [45]. Similarly, otitis and bacterial urinary tract disease are common causes of morbidity in dogs and are among the leading causes of antimicrobial use [46–48]; and surgery often requires antimicrobial prophylaxis and post-operative administration of antimicrobials [49, 50]. The main prescribed antibiotics were cefalexin, amoxicillin, amoxicillin/clavulanic acid, and enrofloxacin. Beta-lactams are commonly the most used antimicrobial group in small animals, especially when culture is not available [12, 14, 47].

Except for penicillinase-labile antibiotics, MSSP usually presents satisfactory rates of antimicrobial susceptibility reported in the literature [10, 51–53]. However, our results revealed high rates of antimicrobial resistance to several classes of antibiotics, including veterinary critically important antimicrobial agents [54], in both MSSP and MRSP strains, with significant occurrence of MDR. In contrast, chloramphenicol and nitrofurantoin were the antibiotics with major rates of susceptibility among *S*. *pseudintermedius* strains, which is expected, since both are forbidden for veterinary use in Brazil [55].

The present study identified an overall occurrence of 24.5% of MRSP among all *S*. *pseudintermedius* isolates, which is within the expected range, since literature reports variable rates of prevalence of MRSP in dogs, ranging from 0% to 60% [3, 9, 56]. Previous studies conducted in Brazil showed similar or higher rates of MRSP in animals with pyoderma, otitis and UTI [22–25, 46] when compared to the present work. It is important to emphasize that comparisons between different studies are not easy to make, as the prevalence of MRSP depends not only on the geographic region but also on the type of sample, the population included, and methodology of the study [1, 57].

Interestingly, over 90% of *S*. *pseudintermedius* strains isolated from surgical wounds were MRSP. Statistical analysis revealed that SSIs, prior antimicrobial treatment, and nosocomial infections are associated with the occurrence of MRSP. This is in agreement with the literature, which has demonstrated that prior antimicrobial exposure, hospitalization, and skin lesions and/or surgical wounds are associated with a higher risk of acquiring MRSP [9, 35, 58–60]. Furthermore, it is possible that the large use of amoxicillin/clavulanic acid, cephalosporins, and fluoroquinolones, which were the main antimicrobials prescribed to dogs included in this study, may be linked to the selection of MRSP [12, 14].

Previous studies using MLST provided information about the population structure of MRSP and have shown that dissemination of this pathogen is linked to some successful clonal lineages, particularly CC71 and CC258 in Europe, CC68 in North America, and both CC45 and CC112 in Asia [56, 61, 62]. In Brazil, only one study has reported the CC of their isolates–a single MRSP ST71 strain isolated from the nostril of a healthy dog [63], so there is no extensive knowledge about the genetic background of *S*. *pseudintermedius* currently circulating in the country. In the present study, approximately 41.7% (10/24) of the MRSP strains belonged to CC71, which was also reported in Brazil previously [63]. Studies have shown that CC71 is frequently resistant to all antimicrobials used in the routine of small animals, including macrolides, aminoglycosides, and fluoroquinolones, and that it can be associated with higher rates of resistance to oxacillin, amoxicillin/clavulanic acid, cephalothin, and ampicillin [4, 21, 64]. In fact, isolates belonging to CC71 in the present study were all resistant to at least six classes of non-beta-lactam antimicrobials, including fluoroquinolones, which are associated with the successful spread of MDR MRSP lineages [53]. Most CC71 strains were isolated from SSIs at the same veterinary hospital (data not shown), which raised the concern of possible nosocomial transmission as previously reported [59]. Further molecular characterization is required to confirm this hypothesis.

Recent studies across Europe, North America, and Oceania have shown that the prevalence of new lineages is increasing, possibly overcoming the prevalence of CC71 within years [4, 5, 35, 62, 65]. In the present study, 62.5% (15/24) of the MRSP strains belonged to nine new STs. All STs were resistant to at least three different classes of non-beta-lactam antimicrobials, and some of them were isolated from more than one animal. This observation corroborates the hypothesis that events of horizontal gene transfer may be as relevant as clonal dispersion to MRSP and highlights the need for more studies in Brazil to better understand the evolution of these new lineages [1, 4, 35, 56].

This is the first study to extensively investigate the phenotypic and molecular characteristics of *S*. *pseudintermedius* isolates in Brazil. In addition, this study identified risk factors for acquisition of MRSP and analyzed the population structure of MRSP strains, which contributes to understanding the dispersion of MRSP not only in Brazil, but also in South America, where there is still a lack of information about it. The present study confirmed that *S*. *pseudintermedius* is the main staphylococcal species isolated from infected dogs in Belo Horizonte. The high rate of MDR observed, especially MRSP-MDR strains, is of great concern. CC71 is currently circulating among Brazilian veterinary clinics and poses a challenge to therapeutic protocols due to its resistance to most antimicrobial classes of daily use in small animal practice and its rapid dispersion. Nevertheless, the new STs found in this study indicate that the spread of MRSP via horizontal gene transfer may be as relevant as clonal dispersion. To our knowledge, this is the first Brazilian report that identified MRSP ST71 in diseased dogs and revealed new circulating STs in the country.

## Supporting information

S1 File Supplementary file: Details of the animals included. (XLSX)

S2 File Raw data of the identification and antimicrobial susceptibility tests. (XLSX)
